# Vitreous levels of interleukin-35 as a prognostic factor in B-cell vitreoretinal lymphoma

**DOI:** 10.1038/s41598-020-72962-z

**Published:** 2020-09-24

**Authors:** Atsunobu Takeda, Eiichi Hasegawa, Shintaro Nakao, Keijiro Ishikawa, Yusuke Murakami, Toshio Hisatomi, Mitsuru Arima, Nobuyo Yawata, Yoshinao Oda, Kazuhiro Kimura, Hiroshi Yoshikawa, Koh-Hei Sonoda

**Affiliations:** 1 Present Address: Department of Ophthalmology, Graduate School of Medical Sciences, Kyushu University, 3-1-1 Maidashi, Higashi-Ku, Fukuoka 812-8582 Japan; 2 Department of Ophthalmology, Chikushi Hospital, Fukuoka University, Chikushino, Fukuoka Japan; 3 Department of Anatomic Pathology, Graduate School of Medical Sciences, Kyushu University, Fukuoka, Japan; 4 Department of Ophthalmology, Graduate School of Medicine, Yamaguchi University, Ube, Yamaguchi Japan; 5 Department of Ocular Pathology and Imaging Science, Graduate School of Medical Sciences, Kyushu University, Fukuoka, Japan; 6 Department of Ophthalmology,Graduate School of Medical Sciences, Kyushu University, Fukuoka, Japan

**Keywords:** Eye cancer, Interleukins, Cancer microenvironment, Prognostic markers

## Abstract

Vitreoretinal lymphoma (VRL) is a rare disease of B-cell origin with poor prognosis. Regulatory cytokines promote tumor development by suppressing antitumor immunity in several cancer types, including B-cell malignancies. To identify the regulatory cytokines associated with poor prognosis in patients with B-cell VRL, we determined the regulatory cytokines profiles in the vitreous humor of patients with VRL. This retrospective study included 22 patients with VRL, 24 with non-infectious uveitis (NIU), and 20 with idiopathic epiretinal membrane (control). Vitreous concentrations of regulatory cytokines were assessed using a cytometric beads assay and association with clinical data was examined. IL-35 and soluble IL-2 receptor α levels were significantly higher in patients with VRL and NIU than those in the control group. The 5-year overall survival (OS) rates for the group with high intravitreal IL-35 was significantly poorer than those for the group with low intravitreal IL-35, who were diagnosed with VRL at the onset (*P* = 0.024, log-rank test). The 5-year OS rates with intravitreal IL-35 levels above and below the median were 40.0% and 83.3%, respectively. Our results suggest that high intravitreal IL-35 levels indicate poor prognosis for patients diagnosed with B-cell VRL at the onset.

## Introduction

The majority of vitreoretinal lymphomas (VRLs) are related to a high-grade non-Hodgkin’s lymphoma (NHL) which is a subset of primary central nervous system lymphoma (PCNSL) that can be subtyped as diffuse large B-cell lymphoma (DLBCL)^1^. VRL often occurs in elderly immunocompetent patients and usually develops in the retina, vitreous chamber, and/or the optic nerve^2^. Most VRLs either present as primary lymphomas, or they occur secondarily to or simultaneously with central nervous system (CNS) lymphomas. In rare cases, VRLs are derived from systemic metastatic lymphomas^1,3^. Primary VRL (PVRL) is a rare form of VRL that is limited to the eye at initial diagnosis; at a referral eye center in Japan, its incidence rate is estimated to be 21 per 100,000 patients with ocular disorders^4^. CNS involvement (an indicator of poor prognosis) develops after the onset of ocular symptoms in 60–90% of PVRL patients^5,6^. Recently, several prospective studies have demonstrated that HD-MTX-based chemotherapy with intravitreal MTX injections could delay the progression of VRL to the CNS, thus improving overall survival (OS)^7–9^. However, it is often difficult to administer these treatments to elderly patients and patients with a poor general condition due to the possibility of treatment-induced adverse events, such as neurotoxicity and nephrotoxicity^10,11^.

The tumor microenvironment (TME) composed of reactive immune cells, stromal cells, blood vessels, and extracellular matrix, regulates tumor cell survival and proliferation, as well as immune evasion for treatment resistance, and is associated with worse prognosis in B-cell malignancies^12^. In PCNSL specimens, tumor-infiltrating lymphocytes (TILs) are found in the perivascular area, accompanied by the accumulation of lymphoma cells around the vessels within the tumor^13^. Recently, several studies have reported that regulatory cytokines in the TME, such as interleukin (IL)-10 and IL-35, support tumor development via the suppression of antitumor immunity in cancer^14–16^. Regulatory T-cells (Tregs), which belong to a highly suppressive subset of T-cells, increase regulatory cytokine secretion for the maintenance of self-tolerance and inhibition of autoimmunity, resulting in tumor development^17,18^. IL-10 is widely regarded and has been analyzed as an immunosuppressive cytokine; it not only exerts inhibitory effects on antitumor immunity by Tregs but is also a potent growth and survival factor for malignant B cells, leading to the exacerbation of B-cell lymphoma^14^. IL-35 is a heterodimeric cytokine, comprising IL-12p35 and Epstein-Barr-induced gene 3 (EBI3) subunits, and is categorized as a IL-12-related cytokine. IL-35 is reported to possess anti-inflammatory and immunosuppressive roles due to its inhibitory effect on the effector T-cell function by the induction of IL-10, and expansion of Tregs and regulatory B cells^19^. IL-35 was also shown to inhibit potent antitumor immunity through the increased expression of inhibitory receptors, such as programmed cell death-1 (PD-1) and T-cell immunoglobulin and mucin domain 3, on TILs in the TME^16^. Furthermore, IL-10-related cytokines, such as IL-22, have been identified in sequence homology search for *IL-10*^20^ and reported to have a regulatory role in ocular inflammatory diseases such as uveitis^21–23^. Thus, the assessment of regulatory cytokines may bring about better comprehension of B-cell VRL pathogenesis, leading to their clinical use, such as in diagnostic markers and therapeutic targets.

In this study, we aimed to reveal the profiles of regulatory cytokines in the vitreous humor of VRL patients, and compare these with those of non-infectious uveitis (NIU) patients. In addition, we assessed the association of regulatory cytokines with clinical parameters in B-cell VRL patients; and observed that higher levels of IL-35 in the vitreous humor were positively correlated with a poor prognosis in B-cell VRL patients with vitreoretinal lesions at initial diagnosis.

## Results

The following VRL patients’ (n = 22) clinical data are presented in Table 1: (1) age at diagnosis of VRL, (2) sex, (3) the eye involved, (4) primary organ involved, (5) presence or absence of HD-MTX-based chemotherapy for CNS prophylaxis or CNS lesions after the initial onset of VRL, (6) presence or absence of HD-MTX-based chemotherapy for the treatment for PCNSL before the initial onset of VRL, (7) brain involvement, (8) relapses after initial diagnosis of malignant lymphoma, and (9) interval between the initial definitive diagnosis of B-cell VRL and death.

**Table 1:** Clinical data of patients with VRL.

Case no.	Sex	Age at diagnosis of B cell lymphoma (year)	Primary origin	Eye involved	HD-MTX-based chemotherapy after vitreoretinal relapse	HD-MTX-based chemotherapy before vitreoretinal relapse	Brain involved	Relapse (mos. after initial diagnosis)	Outcome (mos. after initial diagnosis of vitreoretinal lesions)
1	F	51	Eye	OU	Yes	N/A	Yes	Brain; 16 mos Eye; no relapse	Died 38 mos
2	F	55	Eye	OU	Yes	N/A	Yes	Brain; 80 mos Eye; 20 mos	Alive
3	F	80	Eye	OS	No	N/A	Yes	Brain; 12 mos Eye; no relapse	Died 18 mos
4	M	45	Eye	OU	Yes	N/A	Yes	Brain; 48 mos Eye; 26 mos	Died 83 mos
5	F	73	Eye and Brain	OU	No	N/A	Yes	Brain; 16 mos Eye; no relapse	Died 25 mos
6	F	67	Eye and Brain	OU	Yes	N/A	Yes	Brain; no relapse Eye; no relapse	Alive
7	F	68	Eye	OU	Yes	N/A	Yes	Brain; 68 mos Eye; 52 mos	Died 71 mos
8	M	68	Eye	OU	Yes	N/A	Yes	Brain; 53 mos Eye; 32 mos	Died 70 mos
9	M	68	Eye and Brain	OU	Yes	N/A	Yes	Brain; 20 mos Eye; 20 mos	Died 43 mos
10	F	65	Eye	OU	Yes	N/A	Yes	Brain; 64 mos Eye; 24 mos	Alive
11	F	70	Eye and Brain	OU	Yes	N/A	Yes	No relapse	Alive
12	M	63	Brain	OU	Yes	Yes	Yes	Eye; 10 mos	Died 48 mos
13	M	61	Brain	OU	Yes	Yes	Yes	Eye; 20 mos	Lost to follow-up
14	M	59	Brain	OU	Yes	Yes	Yes	Eye; 33 mos	Lost to follow-up
15	M	38	Brain	OS	Yes	Yes	Yes	Eye; 6 mos	Lost to follow-up
16	F	69	Brain	OU	No	Yes	Yes	Eye; 35 mos	Alive
17	M	66	Brain	OU	Yes	Yes	Yes	Eye; 84 mos	Died 44 mos
18	F	73	Nose, Paranasal sinus	OU	No	Yes	Yes	Eye; 120 mos Brain; 102 mos	Alive
19	M	78	Chest wall	OU	Yes	No	Yes	Eye; 18 mos Brain; 24 mos	Alive
20	F	74	Breast	OU	No	No	No	Eye; 59 mos Brain; no relapse	Died 30 mos., but not due to ML
21	M	68	Testis	OS	Yes	No	Yes	Eye; 108 mos Brain; 96 mos	Died 24 mos
22	F	79	Intestine	OU	No	No	No	Eye; 19 mos	Lost to follow-up

VRL, vitreoretinal lymphoma; F, female; M, male; OU, both eyes; OS, left eye; mos., months; ca., carcinoma; LN, lymph node; N/A, not applicable; ML, malignant lymphoma.

### Vitreous levels of regulatory cytokines

The data for the vitreous levels of regulatory cytokines obtained from patients with controls, VRL, and NIU are summarized in Table 2. IL-10, IL-20, IL-22, IL-27, IL-35, and soluble IL-2 receptor α (sIL-2Rα) levels were significantly different among patients with controls, VRL, and NIU (Table 2; *P* < 0.001, *P* < 0.001, *P* = 0.0011, *P* = 0.026, *P* < 0.001, *P* < 0.001, respectively). Vitreous levels of IL-22 (*P* = 0.0044) and IL-10 (*P* < 0.001) were significantly higher in patients with VRL than those with NIU and controls, whereas none was higher in patients with NIU than those with VRL and the control groups. IL-20 levels (*P* = 0.015) were decreased in patients with NIU compared to those with VRL and the control groups. Vitreous levels of IL-35 (*P* < 0.001) and sIL-2Rα (*P* < 0.001) were increased in patients with VRL and NIU compared to controls, but were comparable in between patients with VRL and NIU. IL-27 levels (*P* = 0.027) were elevated in patients with VRL compared to controls but were comparable to those with NIU. IL-12p40 and IL-26 levels were not significantly different among the three groups. IL-2, IL-12p70, IL-19, IL-28A/IFN-λ2, and IL-29/IFN-λ1 levels in each group were under the detection limit (data not shown).

**Table 2:** Concentrations of regulatory cytokines and sIL-2Rα in the vitreous humor.

	Control (n = 20)	VRL (n = 22)	Uvetis (n = 24)	*P* Value
IL-10 (ng/mL)	0 (0–3.56)	557.9 (525.74–1876.1)^§§, ‡‡^	3.68 (0–15.5)	< 0.001*
IL-12p40 (ng/mL)	33.4 (20.1–39.9)	39.9 (39.9–52.7)	31.7 (26.8–39.9)	0.22^†^
IL-20 (pg/mL)	26.5 (0–86.5)	83.2 (79.9–99.4)^‡‡^	0 (0–16.0)^§,^	< 0.001*
IL-22 (ng/mL)	58.2 (49.3–68.6)	88.9 (84.9–104.4)^§§, ‡‡^	53.3 (38.6–74.5)	0.001*
IL-26 (ng/mL)	90.1 (56.8–161.5)	110.3 (107.9–189.9)	86.0 (51.8–187.3)	0.64^†^
IL-27 (pg/mL)	0 (0–0)	54.4 (54.4–96.0)^§^	5.8 (0–169.1)	0.026*
IL-35 (pg/mL)	59.4 (47.4–75.8)	132.8 (130.0–255.2)^§§^	118.4 (84.7–161.6)^§§^	< 0.001*
sIL-2Rα (ng/mL)	0 (0–0)	260.9 (181.6–690.2)^§§^	205.1 (0–434.8)^§§^	< 0.001*

VRL, vitreoretinal lymphoma; IL, interleukin; s, soluble; R, receptor. Cytokines and sIL-2Rα are expressed as median with interquartile range in parentheses.

*Kruskal–Wallis test.

^†^One-way ANOVA test.

^§^*P* < 0.05: VRL or uveitis vs control.

^§§^*P* < 0.01: VRL or uveitis vs control.

^‡^*P* < 0.05: VRL or control vs uveitis.

^‡‡^*P* < 0.01: VRL or control vs uveitis: Steel–Dwass test or Turkey–Kramer test.

### Elevation of vitreous IL-35 levels may be a prognostic factor in patients with VRL

**Figure 1:**
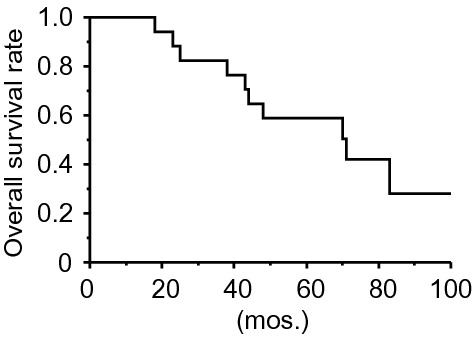
Overall survival curve for 17 patients diagnosed with B-cell vitreoretinal lymphoma.

**Figure 2:**
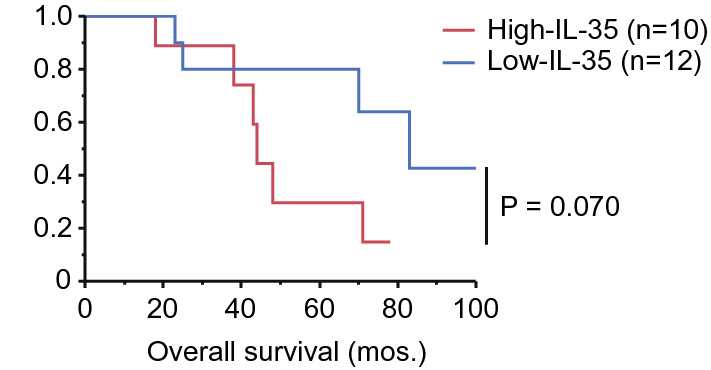
Kaplan–Meier curve of overall survival for patients with B-cell vitreoretinal lymphoma in the high-IL-35 group (≥ 130.0 pg/mL, red line, n = 10) and in the low-IL-35 group (< 130.0 pg/mL, blue line, n = 12) (*P* = 0.070, log-rank test). *P* value < 0.05 was considered significant.

For the 17 patients with B-cell VRL who were followed for more than 5 years, the 5-years OS rate was 58.8% (10/17, Fig. 1). We assessed the association of OS with vitreous levels of regulatory cytokines and sIL-2Rα, the density of CD3^+^ cells in the vitreous humor, age, and sex in patients with VRL. First, to divide 22 patients with VRL into appropriate groups, we divided them into “low” and “high” groups based on whether the above parameters were below or above the median. Among the factors, the OS rates in the high-IL-35 group were lower than those of the low-IL-35 group (non-significant, *P* = 0.070, Fig. 2; the median levels of IL-35 in VRL patients, 130.0 pg/mL). The 5-year OS rates in the high-IL-35 and low-IL-35 groups were 28.6% (2/7) and 80.0% (2/10), respectively.

**Figure 3:**
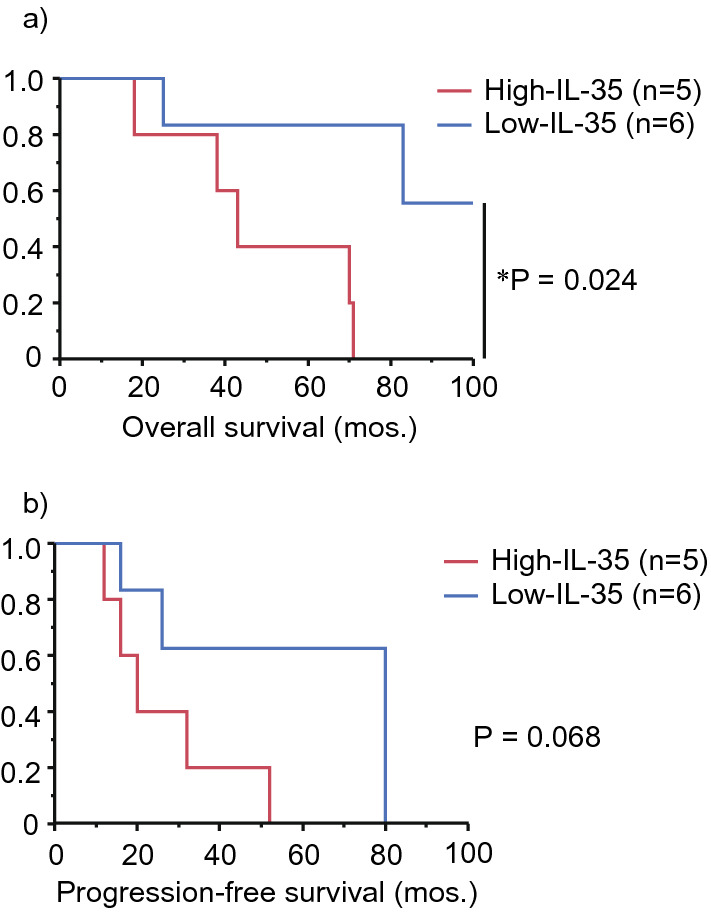
(**a**) Kaplan–Meier curves of overall survival (OS) and (**b**) progression-free survival (PFS) for patients with B-cell primary vitreoretinal lymphoma in the high-IL-35 group (≥ 132.8 pg/mL, red line, n = 5) and for those in the low-IL-35 group (< 132.8 pg/mL, blue line, n = 6) (*P* = 0.024 for OS; *P* = 0.068 for PFS, log-rank test). *P* value < 0.05 was considered significant.

Then, we examined whether the association of OS or progression-free survival (PFS) of patients with primary vitreoretinal lymphoma (PVRL) and VRL with CNS lesions (VRCNSL) that had median levels of IL-35. The OS rates in the high-IL-35 group were significantly decreased compared to those in the low-IL-35 group (*P* = 0.024, Fig. 3A; the median levels of IL-35 in PVRL and VRCNSL patients, 132.8 pg/mL). The 5-year OS rates in the high-IL-35 and the low-IL-35 groups were 40.0% (2/5) and 83.3% (5/6), respectively. In addition, the PFS rates in the high-IL-35 group were lower than those in the low-IL-35 group (non-significant, *P* = 0.068, Fig. 3B). Four of five patients in the high-IL-35 group and five of six patients in the low-IL-35 group were treated with HD-MTX based chemotherapy and/or WBRT for CNS prophylaxis or received the treatment for CNS lesions. The remaining one patient in each group was treated with IV-MTX alone due to their old age and general condition. Neither the OS nor PFS rates of patients with PVRL and VRCNSL showed significant association with other regulatory cytokines, sIL-2Rα, the density of CD3^+^ cells, age, and sex (data not shown).

### The strong correlation of IL-35 and sIL-2Rα levels with the density of CD3^+^ cells in the vitreous body in patients with VRL

**Figure 4:**
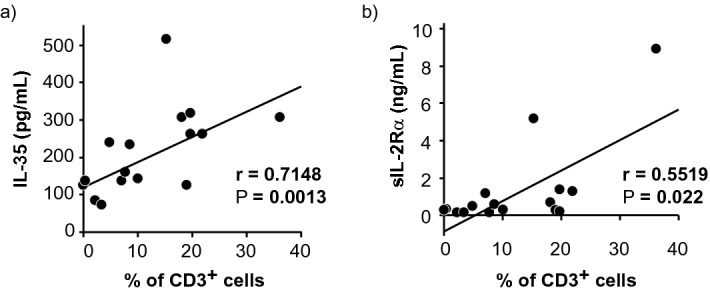
Positive correlations between vitreous concentrations of IL-35 or sIL-2Rα and the density of T-cells in vitrectomy cell blocks from patients with B-cell VRL. (**A**) IL-35 and (**B**) sIL-2Rα (n = 16); r indicates Spearman correlation coefficient. *P* value < 0.05 was considered significant.

Several studies reported that IL-35 is secreted by TIL in the TME^16,24–26^. We previously reported that there is a statistically significant positive correlation between the density of CD3^+^ cells in vitrectomy cell blocks and the vitreous levels of sIL-2Rα in patients with VRL.^27^ Therefore, we examined whether the vitreous levels of IL-35 and sIL-2Rα were related to the density of CD3^+^ cells in vitrectomy cell blocks in patients with VRL (n = 16). There was a statistically significant positive correlation between the density of CD3^+^ cells and the vitreous levels of IL-35 as well as those of sIL-2Rα in patients with VRL (r = 0.7148, *P* = 0.0013; r = 0.5519., *P* = 0.022; Spearman correlation coefficient: Fig. 4A,B).

## Discussion

In this study, we identified differential profiles of regulatory cytokines between patients with B-cell VRL and NIU. The profiles of regulatory cytokines in the vitreous humor showed higher levels of IL-10 and IL-22 in patients with VRL than those in patients with NIU and the controls; and higher levels of IL-35 and sIL-2Rα in patients with VRL and NIU than those with the controls, and higher levels of IL-27 in patients with VRL than those with the controls. In our analysis of regulatory cytokines with clinical parameters, higher levels of IL-35 were indicative of a poor outcome in the vitreous humor at the initial diagnosis of B-cell VRL. In addition, levels of IL-35 and sIL-2Rα were positively correlated with the density of CD3^+^ cells in the vitreous humor in B-cell VRL.

Although responses to HD-MTX-based systemic chemotherapy and/or WBRT for CNS prophylaxis or CNS lesions were effective or led to complete remission (CR) in patients with VRL in this study, the effects of the treatment were limited and not long-lasting. Recently, nivolumab, an anti-PD-1 agent, has been reported to maintain CR in patients with relapsed/refractory PCNSL^28^. PD-1, which is expressed on activated T-cells, such as cytotoxic T lymphocytes (CTLs), interacts with its ligands PD-L1 and PD-L2, which are commonly expressed by tumor cells, leading to enhanced inhibitory signaling of T-cell receptor in CTLs^29,30^. IL-35 can directly upregulate the expression of PD-1 receptors on Tregs and myeloid-derived suppressor cells (MDSCs) to weaken the T-cell mediated antitumor immunity by promoting their inhibitory function and T-cell exhaustion in TIL^16^. This suggests that immune check-point inhibitors, targeting PD-1 or PD-L1, are effective for CNS prophylaxis in patients with PVRL or in patients with recurrent or refractory DLBCL-VRL when IL-35 levels are high in the vitreous humor at the initial diagnosis. In addition, blocking IL-35 is one of the appealing targets to improve prognosis in patients with recurrent or refractory DLBCL-VRL, for whom the concentration of IL-35 is regarded as high in the vitreous humor.

IL-35 is secreted not only by Treg, but also by B cells, monocytes, endothelial cells, and smooth muscle cells at a lesser amount^25^. IL-35 can activate Tregs and convert naïve T-cells into IL-35-producing induced Treg subsets (iTr35 cells), which accumulate in the TME and exert robust suppressive activity^31^. Sawant et al. demonstrated that Treg-derived IL-10 and IL-35 in the TME cooperatively limited antitumor immune responses by regulating the expression of several inhibitory receptors and T-cell exhaustion in CD8^+^-TIL^24^. The histological analysis of breast cancer samples revealed that high IL-35 levels in TILs were significantly associated with advanced cancer stages, resulting in poor PFS and OS^32^. Furthermore, in colorectal cancer patients, IL-35 expression was correlated with the severity of malignancy, cancer clinical stage, and the number of peripheral Foxp3^+^-Tregs^33^. Moreover, IL-35 expression was defined as an independent prognostic factor for recurrence and positively correlated with CD39^+^Foxp3^+^-TILs infiltration in hepatocellular carcinoma^34^. Therefore, these reports together with our own results suggest that Tregs in TIL are the primary source of IL-35 and that IL-35 is involved in the formation of the TME and subsequent tumor progression in patients with B-cell VRL. Furthermore, it is reported that tumor-derived IL-35 promotes MDSCs accumulation in the TME, tumor angiogenesis, and suppression of CTL responses to support tumor growth^19^. In addition, gene expression analysis in DLBCL samples has revealed that increased expression of IL-35 in tumor cells is associated with a worse OS in patients with DLBCL that are treated with systemic chemotherapy^35^. Therefore, our results indicated that higher levels of IL-35 could be induced from DLBCL-VRL cells as well as Tregs in the high-IL-35 group rather than the low-IL-35 group, resulting in the inhibition of antitumor responses. Furthermore, IL-10 and IL-35 augmented their cooperation to limit antitumor immunity by inducing inhibitory receptors, including PD-1, and contributing to T-cell exhaustion of CD8^+^-Tregs once the tumor cells recurred, because IL-10 was abundantly secreted by tumor cells in B-cell VRL.

IL-27 was also reported to play an inhibitory role in antitumor immunity in several types of cancers^36^. IL-27 is a heterodimeric cytokine and one of its subunits, EBI3, is shared with IL-35. IL-27 possesses a proinflammatory or protective role in autoimmune diseases^36^. However, IL-27 was shown to promote CTL and natural killer cell recruitment into the TME, thereby augmenting antitumor immunity^36^. Immunohistological analysis of DLBCL demonstrated that no expression of IL-27p28, another subunit of IL-27, despite the overexpression of both EBI3 and IL-35p35, which are components of IL-35^35^. In our study, IL-27 levels were upregulated but not associated with the prognosis of patients with B-cell VRL, indicating that IL-27 may not be related to the inhibition of the antitumor immunity in B-cell VRL.

Previously, we have shown that sIL-2Rα levels anticipate tissue destruction events caused by the infiltration of T-cells into the neural retina and/or subretina in VRL^27^. In this study, we found that sIL-2Rα levels were not associated with poor OS and PFS in patients with VRL. However, several studies have reported that serum sIL-2Rα levels predict well unfavorable prognosis in systemic DLBCL^37–40^. The complex of sIL-2Rα and IL-2 promotes IL-2-dependent Tregs development and function, and inhibits CD8^+^ T-cells, supporting B-cell lymphoma growth^41^. Recently, Yoshida et al. revealed that sIL-2Rα levels in sera are dependent on the infiltration of tumor-associated macrophages (TAM) into the TME; TAM-derived matrix metalloproteinase-9 plays a pivotal role in the cleavage of IL-2Rα on T-cells in extranodal DLBCL and follicular lymphoma^42^. TAM accumulate within the TME, promoting tumor development, invasion, and metastasis^43^. Overall, our data, aligned with these studies, suggest that sIL-2Rα levels are associated with the infiltration of TAM and CD3^+^ T-cells into the neural retina and/or subretina for promoting tumor invasion, rather than Tregs, which suppress antitumor immunity in VRL.

Th22 cells were shown to be associated with the development and progression of various types of cancers, including B-cell NHL. Th22 cells, which are newly defined subsets of T helper cells, are a source of IL-22 and TNF-α^44^. Recently, IL-22 was found to promote tumor growth in multiple myeloma and mantle cell lymphoma through the aberrant expression of the IL-22 receptor A1^45,46^. In addition, although increased frequency of Th22 cells was associated with a poor response to chemotherapy in B-cell NHL, the frequency of Th22 cells was dissociated with plasma IL-22 levels because IL-22 is produced not only by Th22 cells but also various other types of cells, such as activated T-cells and NK cells^44^. Our assessment of IL-10-related cytokines revealed that the levels of IL-22 were higher in patients with VRL than those of the patients with NIU and the controls, but were not associated with the prognosis of patients with VRL. Thus, these results suggested that increased levels of IL-22 are related to the development of B-cell VRL and that the source of IL-22 is not limited to Th22 cells, resulting in no association between IL-22 levels worse outcome in our study. Further studies are necessary to study a role of Th22 cells in the pathogenesis of B-cell VRL.

There are some limitations to this study. First, because it is a single-center retrospective study, the registration information, patient volume, and variables assessed could not be designed beforehand. A bias of selection may have happened. Second, due to the shortage of samples, the sample size is small. Multicenter studies with a larger sample size may be required to clarify whether IL-35 levels are associated with the prognosis of VRL.

Performing the multiplex bead analyses of vitreous samples in patients with VRL, we determined that IL-35 might suppress antitumor immunity to promote tumor growth in VRL, contributing to poor prognosis of patients with VRL. The potential targeting of immune mediators such as immune check point inhibitors and neutralizing IL-35 warrants further research to develop new diagnostic and treatment strategies.

## Materials and methods

### Patients

This study was conducted in accordance with the guidelines of the Declaration of Helsinki and approved by the Kyushu University Institutional Review Board for Clinical Research. We obtained written informed consent from all the participants before performing any study procedures or examinations.

We examined the eyes of 22 immunocompetent patients with VRL (all classified as DLBCL; 10 men and 12 women; mean age, 65.4 ± 10.6 years), including 11 patients with PVRL and VRL with concurrent CNS lesions (VRCNSL), and 11 patients with secondary VRL (6 patients from PCNSL, one patient from testis, and four patients from systemic lesions except for CNS and testis). We also examined 24 patients with NIU (7 men and 17 women; mean age, 62.0 ± 9.88 years) and 20 patients with idiopathic epiretinal membrane (ERM) (7 men and 13 women; mean age, 66.3 ± 7.55 years) as negative controls at the Department of Ophthalmology, Kyushu University Hospital, between January 2008 and December 2019. All patients were Asian adults. VRL confined to the eyes at initial diagnosis was defined as PVRL. VRL accompanied by CNS lesions at initial diagnosis was defined as VRCNSL. Secondary VRL was defined as SVRL. The diagnosis of patients with VRL was based on the definitive identification of malignant lymphoid cells in the eye using either cytological smear or vitrectomy cell block technique^47^.

The patients with preoperative trauma, pre-existing macular pathologies, vitreous hemorrhage, and diabetes mellitus, which are likely to affect the immune mediators in the vitreous humor, were excluded from the study.

### Measurement of regulatory cytokines and the density of CD3^+^ cells

After diluting the above-mentioned supernatants collected from the vitreous body with 10 × phosphate buffered saline, the concentration of each of the following regulatory cytokines was measured using Luminex 100 (Luminex, Austin, TX, USA) according to the manufacturer’s directions: Human regulatory cytokines; IL-2, IL-10, IL-12 (p40), IL-12 (p70), IL-19, IL-20, IL-22, IL-26, IL-27 (p28), IL-28A/IFN-λ2, IL-29/IFN-λ1, and IL-35 using Biorad Human T-reg-plex (Temse, Belgium) and sIL-2Rα using Milliplex MAP Human Soluble Cytokine Receptor Panel (St. Charles, MO, USA). Levels of regulatory cytokines and sIL-2Rα were obtained from the standard curves for each. When the concentrations were below the detection limit, they were coded as zero and were included in the statistical analysis.

When investigating the correlation of regulatory cytokines with the density of CD3^+^ cells, we used the data we had reported previously for the density of CD3^+^ cells^27^.

### Statistical analysis

The data were analyzed using Jump software version 14 (Business Unit of SAS, Cary, NC). If the data were normally and equally distributed, a one-way analysis of variance (ANOVA) was used to compare the vitreous concentrations of each cytokine among the groups, followed by Turkey–Kramer test to detect significant differences between VRL, NIU, and controls. If the data were not distributed normally or equally, the Kruskal–Wallis test was performed to compare the vitreous levels of each regulatory cytokines and sIL-2Rα, followed by Steel–Dwass test to detect significant differences between the three groups. Two-group comparisons of numerical variables were performed using the Mann–Whitney’s *U *test. OS was measured from the time of definitive diagnosis of VRL to death by any cause. PFS was measured from the time of definitive diagnosis of VRL to relapse or death by DLBCL. Patients with VRL were divided into two groups based on the median vitreous levels of each regulatory cytokine and sIL-2Rα. The Kaplan–Meier method was used to estimate the survival curves for each vitreous levels of regulatory cytokines and sIL-2Rα, the density of CD3^+^ cells in the vitreous humor, age, and sex, and the differences between the curves were evaluated by the log-rank test. To determine a correlation between T-cell density and levels of IL-35 or sIL-2Rα, Spearman correlation tests were used. *P* value < 0.05 was considered to be significant.

## Data Availability

The datasets generated during and/or analyzed during the current study are available from the corresponding author on reasonable request.
